# Cilgavimab and tixagevimab as pre-exposure prophylaxis in vaccine non-responder kidney transplant recipients during a period of prevalent SARS-CoV-2 BA.2 and BA.4/5 variants—a prospective cohort study (RESCUE-TX)

**DOI:** 10.1016/j.ebiom.2024.105417

**Published:** 2024-10-22

**Authors:** Roman Reindl-Schwaighofer, Andreas Heinzel, Lukas Raab, Robert Strassl, Carsten T. Herz, Florina Regele, Konstantin Doberer, Oliver Helk, Paul Spechtl, Constantin Aschauer, Karin Hu, Rahel Jagoditsch, Bianca Reiskopf, Georg A. Böhmig, Bernhard Benka, Benedikt Mahr, Karin Stiasny, Lukas Weseslindtner, Michael Kammer, Thomas Wekerle, Rainer Oberbauer

**Affiliations:** a Division of Nephrology and Dialysis, Department of Medicine III, Medical University of Vienna, Vienna, Austria; b Department of Laboratory Medicine, Division of Clinical Virology, Medical University of Vienna, Vienna, Austria; c Austrian Agency for Health and Food Safety (AGES), Vienna, Austria; d Astra Zeneca Medical, Vienna, Austria; e Center for Virology, Medical University of Vienna, Vienna, Austria; f Center for Medical Data Science, Institute for Clinical Biometrics, Medical University of Vienna, Vienna, Austria; g Division of Transplantation, Department of General Surgery, Medical University of Vienna, Vienna, Austria

**Keywords:** COVID-19, Kidney transplant, Vaccine non-responder, Cilgavimab/tixagevimab

## Abstract

**Background:**

The response to severe acute respiratory syndrome coronavirus type 2 (SARS-CoV-2) vaccination is severely impaired in patients on maintenance immunosuppression after kidney transplantation.

**Methods:**

We conducted a prospective cohort study of 194 kidney transplant recipients (KTR) who exhibited no response to SARS-CoV-2 vaccinations (i.e., SARS-CoV-2 spike protein antibodies ≤264 U/mL) and had no prior documented infection. Patients received 300 mg of cilgavimab/tixagevimab as SARS-CoV-2 pre-exposure prophylaxis (PrEP) between March 4, 2022, and May 3, 2022 and were contrasted to a matched cohort of 186 KTRs also without immunization again defined as SARS-CoV-2 spike protein antibodies ≤264 U/mL and no documented prior infection. The primary outcome was the serum kinetics of cilgavimab/tixagevimab, the secondary endpoints were time to SARS-CoV-2 breakthrough infection, severity of disease and variant specific live viral *in vitro* neutralization tests of patient sera.

**Findings:**

Longitudinal serum level monitoring showed a half-life of 91 days for both antibodies (95% CI 86–95 days for cilgavimab and 85–96 days for tixagevimab) in KTRs. *In vitro* neutralization tests showed effectiveness against the BA.2 omicron subvariant but not BA.5. The cumulative incidence of SARS-CoV-2 infections until May 15, 2022, (BA.2 dominance) was 15/194 vs 36/186 in the PrEP and control group respectively (OR = 0.35, 95% CI 0.18–0.66) but was not different thereafter (BA.4/5 dominance). The number of severe infections during the BA.2 period was lower in the prophylaxis than in the control group (OR = 0.37, 95% CI 0.17–0.79).

**Interpretation:**

This study showed that SARS-CoV-2 PrEP with cilgavimab/tixagevimab demonstrated clinical effectiveness against variants that are neutralised (BA.2) but not against BA.4/5.

**Funding:**

This study was funded by the 10.13039/501100005788Medical University of Vienna and an unrestricted grant from 10.13039/100004325AstraZeneca (ESR-21-21585).

**Research in context**

**Evidence before this study**

Maintenance medical immunosuppression is mandatory in patients after kidney transplantation to prevent an alloimmune response but at the same time reduces immunization efficacy after vaccinations. Roughly one third of kidney transplant recipients remained without protection against coronavirus disease 2019 (COVID-19) due to severely impaired immunization after vaccination. During the peak of the COVID-19 pandemic in March of 2022, the modified dual humanised antibody combination cilgavimab/tixagevimab, which targeted two epitopes of the severe acute respiratory syndrome coronavirus type 2 (SARS-CoV-2) spike protein, was the only approved pre-exposure prophylaxis (PrEP) in vaccine non-responders. Given the fast mutation rate of the SARS-CoV-2 strains and especially the omicron variants it was unclear after European Medicines Agency (EMA) approval on March 25, 2022, whether the PrEP with cilgavimab/tixagevimab was effective in prevention of infection and modification of disease severity. Furthermore, the serum half-life of the cilgavimab/tixagevimab combination, which exhibits a modified Fc fragment to extend effectiveness, remained unknown in patients after kidney transplantation who naturally exhibit reduced kidney function. Finally, it was unclear if *in vitro* live-virus neutralization tests correlate with the clinical effectiveness in community-dwelling kidney transplant recipients during a pandemic.

**Added value of this study**

We provide evidence for the correlation of *in vitro* effectiveness of a PrEP combination against SARS-CoV-2 and clinical outcome in immunocompromised transplant patients: *In vitro* neutralization assays over 24 weeks following cilgavimab/tixagevimab administration showed strong neutralization capacity in patient sera against the omicron BA.2 variant but not against BA.5. In line, PrEP using cilgavimab/tixagevimab was effective against SARS-CoV-2 infection during the BA.2 but not the subsequent BA.4/5 wave, respectively. The added value of this study is that it shows that *in vitro* effectiveness data may be used to guide the choice of PrEP in patients at risk.

**Implications of all the available evidence**

Our data showed that the serum kinetics of the Fc-modified antibodies in kidney transplant patients with reduced kidney function was similar to the general population. Furthermore, PrEP using cilgavimab/tixagevimab can effectively reduce SARS-CoV-2 infection of viral strains that are efficiently neutralised, thereby preventing COVID-19 disease in a risk population of immunosuppressed kidney transplant recipients without previous immunological response to vaccination. However, due to continuous mutations of the SARS-CoV-2 spike protein, efficacy of antibody combinations must be continuously re-evaluated as escape variants significantly reduce clinical efficacy.

## Introduction

During the severe acute respiratory syndrome coronavirus type 2 (SARS-CoV-2) pandemic, immunosuppressed patients were found to be a particularly vulnerable group with increased complication rates and mortality compared to non-immunosuppressed individuals.^1^ Recipients of solid organ transplants, patients with malignancies of solid organs or the hematopoietic system, persons with autoimmunological diseases, or acquired immunodeficiency syndrome patients have a reduced humoral and/or cellular immune response due to the therapeutic use of immunosuppressants, systemic glucocorticoids, anti-inflammatory biologics or cytostatic drugs, and disease-intrinsic factors, which are associated with an increased susceptibility to infection and complications.^2^^,^^3^ Since the beginning of 2021, mRNA- and viral vector-based vaccines have effectively protected large segments of the population against coronavirus disease 2019 (COVID-19), particularly its severe disease manifestations.^4^ However, as expected, immunosuppressed individuals showed a reduced vaccine response rate as measured by concentrations of antibodies against the SARS-CoV-2 spike protein, which was related to the choice and dose of immunosuppressive regimens.^5^, ^6^, ^7^, ^8^

Monoclonal antibodies against SARS-CoV-2 were initially developed for treatment of COVID-19, but have since been used in immunocompromised individuals as pre-exposure prophylaxis (PrEP). In the beginning of 2022, the omicron substrain BA.2 replaced BA.1 as the dominant variant in Austria accounting for 62% of all cases in early March of 2022.

At study initiation in March of 2022, cilgavimab/tixagevimab (Evusheld®, AstraZeneca) remained the only monoclonal antibody combination approved for PrEP against the omicron variant in vaccine non-responder transplant patients. Evusheld® received an emergency use authorization from the U.S. Food and Drug Administration (FDA) on December 8, 2021, and from the European Medicines Agency (EMA) on March 25, 2022. Sotrovimab on the other hand, which received only authorization for treatment of COVID-19, was no longer authorised in the USA by the Centre for Disease Control and Prevention (CDC) as of April 5, 2022, because of its ineffectiveness against the prevalent BA.2 omicron subvariant.

In this paper we report the result of the RESCUE-TX study (Recombinant SARS-CoV-2-antibodies in kidney transplant recipients without neutralizing antibody response following full vaccination), a prospective single-centre trial of 194 kidney transplant patients receiving SARS-CoV-2 PrEP to assess serum kinetics of cilgavimab/tixagevimab in kidney transplant recipients over 48 weeks and *in vitro* neutralization capacity of patients’ sera against the dominant SARS-CoV-2 variants (i.e., omicron BA.2 and BA.5). Further, we studied the clinical effectiveness compared to matched vaccine non-responder kidney transplant recipients without prophylaxis over 24 weeks stratified for the dominant variant at time.

## Methods

### Study design

This was an investigator-initiated, single centre prospective cohort study conducted from December 3, 2021, to April 25, 2023. The main endpoint were serum levels of cilgavimab/tixagevimab as PrEP over 48 weeks in kidney transplant recipients. From December 2021 to May 2022 316 adult kidney transplant recipients were screened for inclusion into the study. Inclusion criteria were (1) no humoral response to at least two SARS-CoV-2 vaccinations (defined as SARS-CoV-2 spike protein antibodies ≤264 U/mL^8^^,^^9^) and (2) no documented prior infection with SARS-CoV-2. A total of 194 kidney transplant recipients were enrolled into the study and received SARS-CoV-2 PrEP with cilgavimab/tixagevimab between March 4, and May 3, 2022, in the clinic of the Department of Nephrology and Dialysis at the Medical University of Vienna (Austria). For each monoclonal antibody 150 mg were injected into the left and right deltoid muscle.

The clinical follow up was 24 weeks, during which all SARS-CoV-2 positive cases were obtained via the centralised AGES (Austrian Agency for Health and Food Safety) repository which holds all SARS-CoV-2 PCR test results from the population of Austria. The underlying SARS-CoV-2 variant was identified by the Division of Clinical Virology of the Medical University of Vienna.

**Fig. 1:**
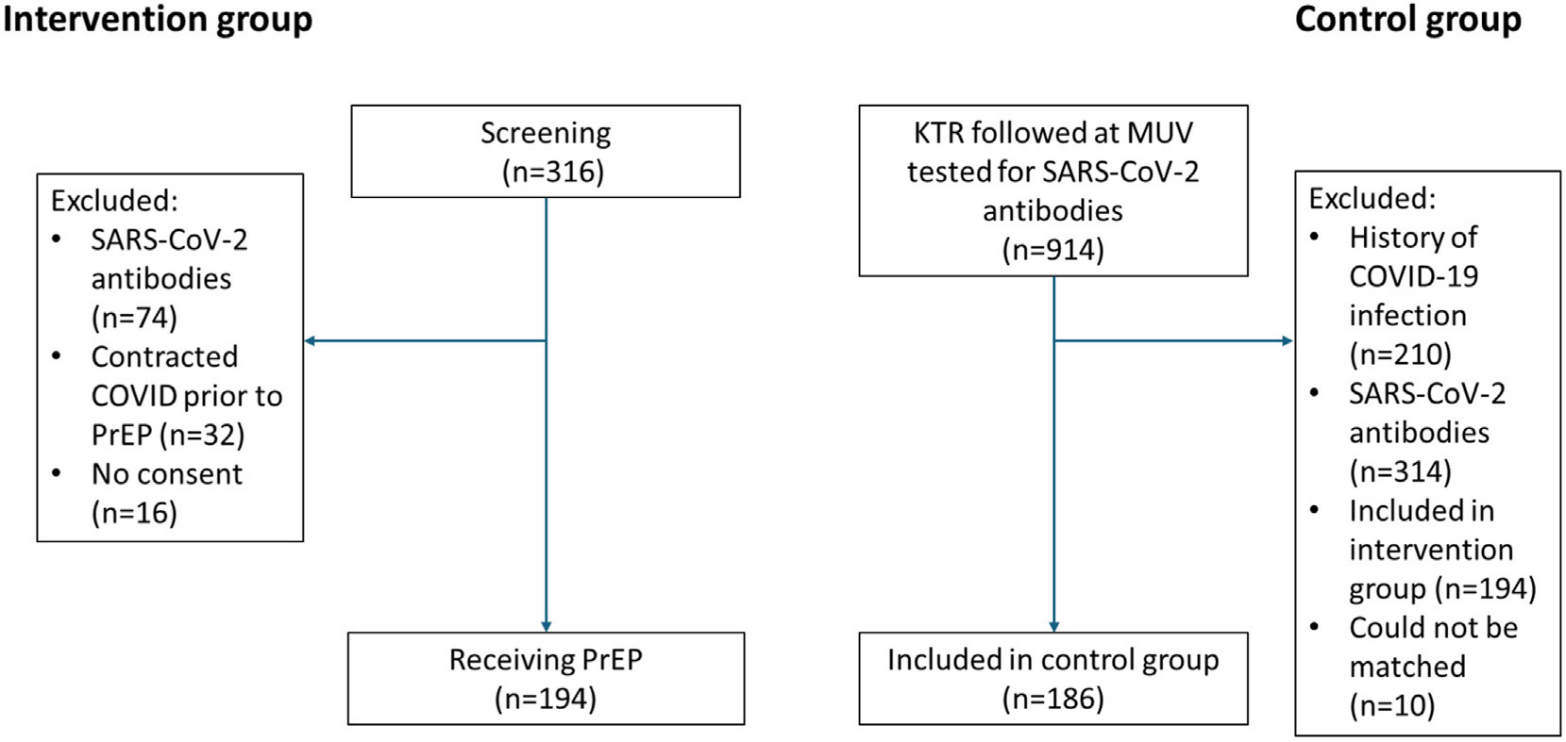
Study recruitment flow chart: The screening of 316 patients was required to enroll the 194 PrEP patients. The control group was assembled from all kidney transplant recipients attending our outpatient clinic in the months prior and during the recruitment phase who had a current SARS-CoV-2 antibody level measurement (n = 914). Of those, 196 patients fulfilled the inclusion criteria of the study (SARS-CoV-2 spike antibody levels ≤264 U/mL and no documented prior infection) and were not part of the PrEP group. These patients were paired 1:1 with patients in the PrEP group in order to synchronize the begin of observation time between the two groups. For ten of the 196 potential control patients no appropriate partner could be identified and thus these had to be excluded. The remaining 186 control patients who could be successfully paired with a PrEP patient formed the control group.

The control group was selected from 914 kidney transplant recipients with SARS-CoV-2 antibody levels measured during an appointment in our outpatient clinic within 90 days before the date of dosing of any of the study participants. Control patients without immunization, defined by the same inclusion criteria as for PrEP patients, were paired 1:1 with PrEP patients to synchronise the start of observation for the time to event analysis, i.e., to ensure a common time zero. For the intervention group time zero was the date of PrEP application. In detail, PrEP patients were ordered by screening date and for each PrEP patient a set of all eligible control patients was determined. A patient had to fulfill the following criteria to be added to the set: (1) was not part of the intervention arm of this study, (2) was not selected as control patient for any of the prior PrEP patients, (3) received a kidney transplant before the date of dosing of the current PrEP patient, (4) did not contract a SARS-CoV-2 infection before the date of dosing of the current PrEP patient, (5) had one or more SARS-CoV-2 antibody tests within 90 days before dosing of the current PrEP patient and none of these SARS-CoV-2 antibody tests exceeded 264 U/mL. From this set of potential control patients, the patient with the smallest difference in transplant age compared to the current PrEP patient entered the control group of the study and the control patients’ time zero was set to the PrEP patient’s time of PrEP application. The approach employed here of synchronizing the exposure time in vaccine recipients and their paired controls has also been used in a previous study.^10^ Fig. 1 provides a flow chart of patient inclusion. All study results were reported according to the Strengthening the Reporting of Observational Studies in Epidemiology (STROBE) guidelines. Women were enrolled on an equal basis and the sex distribution of one third females correspond to the lower rate of female kidney transplant recipients worldwide. All participants were from European ancestry/Caucasian ethnicity according to the European background population.

The full study protocol can be found at https://www.meduniwien.ac.at/nephrogene/trials/RESCUE-TX/BOOST-TX_RESCUE-TX_protocol_v3.2.pdf as requested.

### Ethics

The trial was registered in the European Union Clinical Trial Register (EudraCT 2021-002927-39) and was approved by the ethics committee of the Medical University of Vienna (No. 1612/2021 and 1174/2022 for the control group) as well as by the competent national authority AGES. The sample size was determined by the number of available PrEP doses at that time of the pandemic.

The study was conducted in accordance with the principles of the Declaration of Helsinki and was sponsored by the Medical University of Vienna and an unrestricted grant from Astra Zeneca, who provided the study drug free of charge. All study participants signed an informed consent before study entry.

### Outcomes

The primary outcome was the serum concentration of cilgavimab and tixagevimab at two, four, eight, 12, 24, 36 and 48 weeks after injection. Secondary endpoints were time to SARS-CoV-2 breakthrough infection and severity of disease as well as variant specific neutralization tests of patient sera *in vitro* using live viral omicron variants BA.2 and BA.5. A complete history of SARS-CoV-2 infections for both the prospective trial population as well as the control group was generated by merging study patient data and the national AGES SARS-CoV-2 registry.

### Measurements

Antibody serum concentrations over time were measured by PPD diagnostics (PPD Development, Chicago, IL, USA), by LC/MS/MS analysis for the detection of cilgavimab and tixagevimab. An Anti-SARS-CoV-2 enzyme immunoassay (Elecsys®, Roche Diagnostics, CH), which tests for the receptor-binding domain of the SARS-CoV-2 spike protein was used to assess anti-spike protein antibodies at screening. Neutralizing antibodies were determined with *in vitro* live-virus neutralization tests using omicron BA.2 sublineage GISAID BA.2:EPI_ISL_11110193 and BA.5 sublineage BA.5.3:EPI_ISL_15982848 as described previously.^11^^,^^12^ SARS-CoV-2 infections were defined as a positive RT-PCR result. During the pandemic, a widespread free of charge national PCR testing service for SARS-CoV-2 was available to everybody (including uninsured persons) in Austria. Results were centralised at AGES. Those study participants who tested positive provided an additional sample for identifying the underlying SARS-CoV-2 variant using RT-PCR melting-curve VirSNiP Mutations Assays (TIB MOLBIOL, Berlin, Germany) performed at the Division of Clinical Virology of the Medical University of Vienna.

Severity of the infection was graded as asymptomatic (positive SARS-CoV-2 PCR at routine testing), symptomatic (any kind of symptom but not requiring hospitalization), hospitalization for SARS-CoV-2, ICU admission and death. For the trial population severity of SARS-CoV-2 infections was assessed at the scheduled study visits and for control group patient´s severity of disease was collected from medical records and telephone interviews.

### Statistics

Demographics of the study participants were summarised as mean and standard deviation for continuous variables and absolute and relative frequencies for categorical parameters. Differences in baseline demographics between the two cohorts were assessed by t-test for continuous variables and chi-squared test for categorial variables. The distribution of the time to breakthrough infection was estimated by the Kaplan–Meier estimator separated for the two eras of the prevalent strains BA.2 and BA.5 and differences between the PrEP and control groups were assessed using a log rank test. For the first era, time to breakthrough infection was calculated starting from the day of PrEP administration until infection or end of the BA.2 era (May 15, 2022). For the second era, time to breakthrough infection was calculated starting from May 16, 2022, until infection or end of observation period (August 15, 2022). Patients were censored in case of non-COVID-19 related death or at the end of the respective era. In addition to the primary analysis of the full study cohort the analysis was repeated in only the subset of paired patients using a stratified log-rank test to account for the pairing-procedure. Antibody levels at study visits were summarised as geometric means and the geometric standard deviation was used to describe their dispersion. A linear model was employed to estimate the association of patient characteristics with peak antibody levels at the two weeks visit. The serum half-life of cilgavimab and tixagevimab was estimated using linear mixed models with B-splines for longitudinal data with patient specific random intercepts, using observation time as single independent variable. We performed visual inspection of residual plots (fitted values vs residuals) to check the linearity assumption and heteroscedasticity, as well as residual quantile–quantile plots to check for normality and determined that the model fit was adequate. A confidence interval for the serum half-life was estimated using individual based bootstrap resampling with 500 redraws. Any missing antibody measurements due to non-availability of a sample for a study participant at a specific visit were considered missing at random and thus no imputation methods were employed. To check for potential confounding, we extended the linear mixed model by including all clinical variables provided in Table 1 as main effects and in interaction with observation time. The numbers of symptomatic COVID-19 infections between groups were quantified by odds ratio (OR) and 95% confidence interval (CI). Odds ratios were primarily calculated by unconditional maximum likelihood estimation. Further, adjusted odds ratios were estimated using generalised linear models including all clinical variables from Table 1 as additional independent variables. Due to the low number of events, a Fisher’s exact test was employed to test for differences in the number of deaths between groups. A p-value below 0.05 was considered statistically significant, no adjustment for multiplicity was performed. All statistical analyses were performed with R version 4.1.2.

**Table 1:** Baseline characteristics of study and control cohort.

	Cilgavimab/tixagevimab N = 194	Control N = 186	p-values
Female (count, %)	72 (37)	73 (39)	0.747
Male (count, %)	122 (63)	113 (61)	
Age (mean, SD) years	62 (11)	57 (15)	<0.001
BSA (mean, SD) m^2^	1.87 (0.21)	1.86 (0.22)	0.468
Time since transplant (mean, SD) years	7 (6)	8 (7)	0.128
Creatinine at inclusion (mean, SD) mg/dL	1.8 (1.2)	1.9 (1.4)	0.730
CNI based triple immunosuppression (count, %)	160 (82)	167 (90)	0.052
Costimulation blocker (count, %)	20 (10)	7 (4)	0.022
Comorbidities (count, %)			
COPD	18 (9)	13 (7)	0.531
CVD	53 (27)	51 (27)	1
DM	65 (34)	54 (29)	0.407
aHT	159 (82)	140 (75)	0.140
CMP	15 (8)	20 (11)	0.400

Body surface area (BSA), Calcineurin inhibitors (CNI), Chronic obstructive pulmonary disease (COPD), cardiovascular disease (CVD), Diabetes mellitus (DM), aHT (Arterial hypertension), CMP (Cardiomyopathy).

### Role of funders

This study was funded by the Medical University of Vienna and an unrestricted grant from AstraZeneca (ESR-21-21585). The funders had no role in study design, data collection, data analyses, interpretation, or writing of the report.

## Results

### Participant characteristics

The demographic characteristics of the study population may be found in Table 1. In brief the PrEP and control cohort consisted of roughly 60% males as usual in kidney transplantation. The median age was around 60 years and PrEP and controls participants received their transplant seven to eight years prior to study onset. It is of note that all participants were on triple maintenance immunosuppression, few with costimulation blockade. Less than 10% had a known history of chronic obstructive pulmonary disease (COPD) and about one third of the participants exhibited diabetes mellitus.

### Serum kinetics of cilgavimab and tixagevimab in kidney transplant recipients

None of the patients had detectable cilgavimab and tixagevimab levels prior to receiving PrEP. The geometric means of peak serum levels of cilgavimab and tixagevimab observed at two weeks after PrEP were 15.9 and 17 μg/mL (SD 1.3 and 1.4 μg/mL), respectively. Concentrations of both antibodies declined in the following weeks and reached 0.7 and 0.9 μg/mL (SD 2.4 and 2.5 μg/mL) at 48 weeks after PrEP, respectively. The geometric mean and SD of the antibody levels for all time points are indicated in Supplementary Table S1. Age, creatinine, body surface area (BSA), and diabetes mellitus were associated with the peak serum levels at the two weeks visit (Supplementary Fig. S1).

**Fig. 2:**
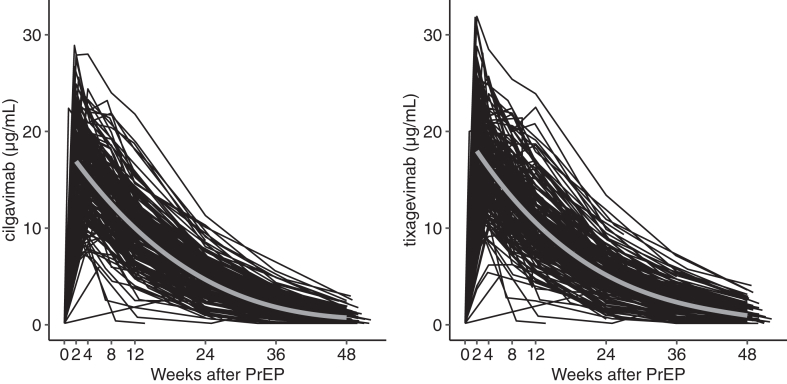
Serum concentration of cilgavimab (left panel) and tixagevimab (right panel) over 48 weeks. The grey line indicates a smoothed estimate for concentration levels from linear mixed models, starting at the peak concentration level two weeks after PrEP.

The half-life was calculated as 91 days for both cilgavimab and tixagevimab, (95% CI 86–95 days for cilgavimab and 95% CI 85–96 days for tixagevimab (Fig. 2). Inclusion of clinical covariables did not change the predicted antibody levels over time (Supplementary Fig. S2).

### Neutralization capacity of patient sera against dominant variants

**Fig. 3:**
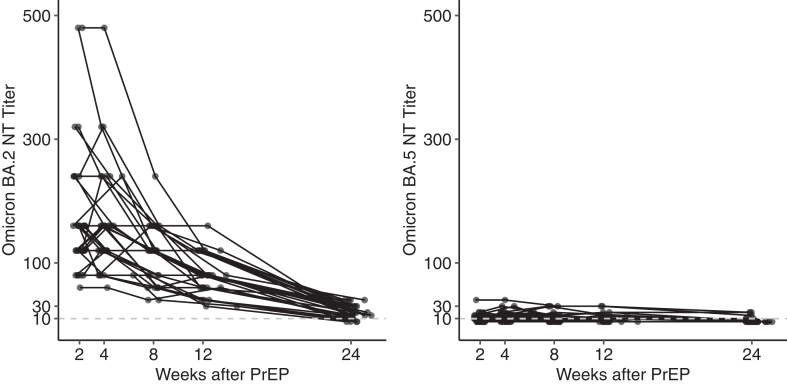
Variant specific live-virus neutralization tests (NT) of patient sera for BA.2 (left panel) and BA.5 omicron (right panel) subvariants at two, four, eight, 12 and 24 weeks after PrEP. The dashed (grey) line indicates the *in vitro* efficacy threshold of NT titers ≥10.

SARS-CoV-2 omicron BA.2 and BA.5 neutralization tests with sera from a subgroup of 30 randomly selected patients confirmed strong activity against BA.2 for 12 weeks after PrEP but was low at week 24. BA.5 neutralization activity in patient sera was severely reduced at all time points (Fig. 3).

### Clinical effectiveness of PrEP

**Fig. 4:**
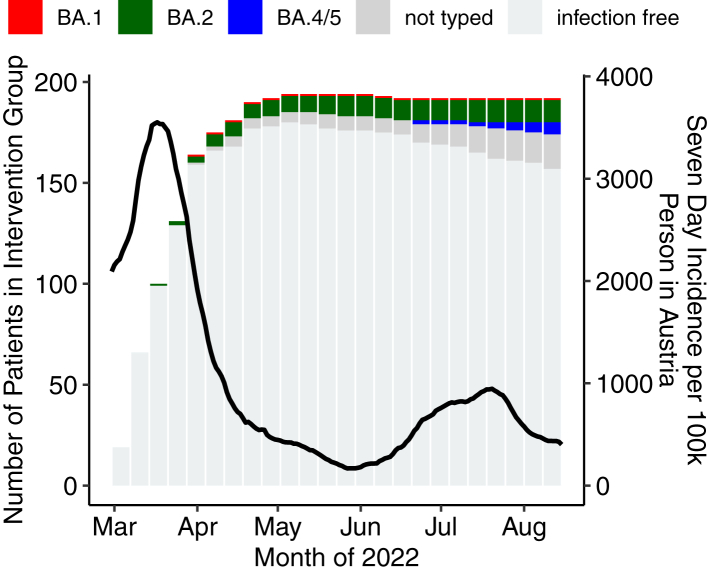
Cumulative infections in patients with cilgavimab/tixagevimab prophylaxis against the background infection rate (black line) in the general population of Austria during the two omicron variant periods BA.2 (until mid May) and BA.4/5 (mid August).

**Fig. 5:**
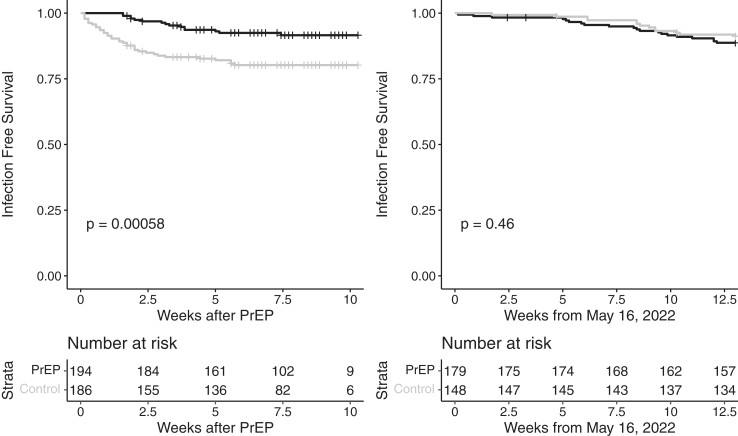
Event-free survival from breakthrough infection for the study cohort and control group during the era of BA.2 prevalence (until May 15) (left panel) and during the BA.4/5 period (May 16-August 15) (right panel). The p-value for a difference between groups was derived using a log-rank test. A stratified log-rank test accounting for the pairing-procedure yielded a p-value of 0.0003 for the first era. Note: The noticeable drop in patients at risk after 7.5 weeks in the left figure is due to the right censoring at the end of the era of BA.2 prevalence. As patients were recruited over roughly two months their available follow up until this date varied hence causing the number of patients at risk in the Kaplan–Meier analysis to decrease.

The cumulative incidence of SARS-CoV-2 infections over the entire follow up period of 24 weeks was 35/194 in the PrEP group and 49/186 in the control group (OR = 0.62, 95% CI 0.38–1.00, p = 0.0512). Fig. 4 shows the cumulative number of infections in the study population against a background of the overall infection rate in the general population that was predominantly driven by two viral strains (i.e., BA.2 until mid-May and subsequently BA.4/5), see Supplementary Table S2. However, the infection incidence stratified for the dominant variant at the time, i.e., BA.2 until mid-May, was 15/194 vs 36/186 in the PrEP and control group over 12 weeks, respectively (OR = 0.35, 95% CI 0.18–0.66, p = 0.0009). In the subsequent time period from May 16 to August 15, 2022 (BA.4/5 dominance) the incidence of SARS-CoV-2 infection did not differ between the groups (20/179 vs 13/148, OR = 1.31, 95% CI 0.63–2.72, p = 0.475). The time-to-event analyses for the two eras are provided in Fig. 5.

**Fig. 6:**
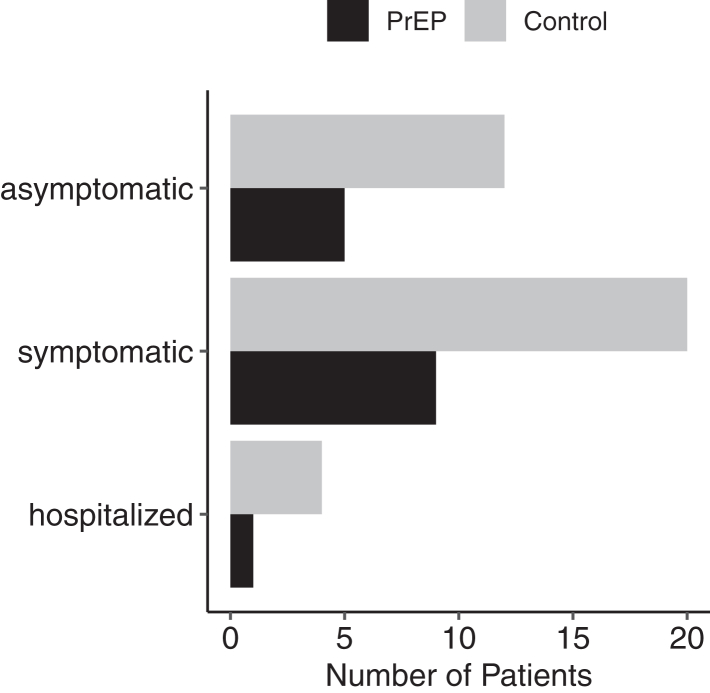
Severity of infections during BA.2. During the BA.2 period until May 15, 15 patients in the PrEP group and 36 in the control group were SARS-CoV-2 PCR positive. Overall, statistically less patients in the PrEP group experienced a severe infection or required hospital admission (OR = 0.37, 95% CI 0.17–0.79, p = 0.0082). No difference was observed thereafter during the BA.4/5 wave.

Overall, statistically significant less patients in the PrEP group developed symptomatic diseases or required hospital admission during BA.2 dominance (OR = 0.37, 95% CI 0.17–0.79, p = 0.0082) (Fig. 6). No difference was observed during the BA.4/5 wave (OR = 0.96, 95% CI 0.43–2.15, p = 0.924). Summary of OR and additionally adjusted OR for efficacy outcomes of PrEP are provided in Supplementary Table S3. During the clinical observational period of 24 weeks, two patients died in the PrEP group and five in the control group (p = 0.275).

## Discussion

This study showed in a cohort of kidney transplant vaccine non-responders that the pharmacokinetic, i.e., the serum half-life of the two monoclonal antibodies directed against the anti-SARS-CoV-2 spike codon was 91 days each and thus similar to healthy volunteers.^13^^,^^14^ Peak serum levels were associated with age, creatinine, diabetes mellitus and BSA. We furthermore showed that the effectiveness of the cilgavimab/tixagevimab correlates with the *in vitro* neutralization capacity of patient sera. PrEP was effective for prevention of BA.2 infections but not against BA.4/5.

A group from Rouen, France reported the efficacy of cilgavimab/tixagevimab PrEP in a cohort of kidney transplant patients and found it effective against BA.2 and of unclear effectiveness against BA.5 as only two BA.5 infections were observed. However, the authors did not test for *in vitro* efficacy against this subvariant.^15^^,^^16^ The same authors showed earlier that PrEP may be effective against BA.2 but not against BA.1.^15^

Colleagues from Boston showed that PrEP with the two antibodies applied during the omicron wave was effective in solid organ transplant patients, by investigating 114 patients with kidney grafts who had been vaccinated before. However, no data on the serum conversion and strength of immunization after the vaccination was reported.^10^ An interesting observation in this paper was also that the later recommended double dose, i.e., 2 × 300 mg of both antibodies applied to little over half of the cohort were more effective than the 2 × 150 mg which were initially recommended by the manufacturer. The median follow-up of the kidney transplant patients was almost three months and fortunately only few infections occurred in both groups.

Other investigators could not confirm the observation of a benefit of prophylaxis with the higher dose. Glhoom and colleagues conducted a systematic review of papers published before December 31, 2022, on PrEP with cilgavimab/tixagevimab in immunocompromised patients and concluded that no relevant variation between the high and low doses was found.^17^ However, given the limited quality of some of the included observational studies the dosing recommendation for transplant patients is still to be determined and may also depend on the prevalent omicron subvariant.

Another study from France investigated the effectiveness of 2 × 150 mg cilgavimab/tixagevimab PrEP against omicron BA.1 and BA.2 in 1112 immunocompromised patients including 511 kidney transplant recipients with impaired vaccine response during the first three months of 2022.^18^ The prevalent variants at this time were omicron BA.1 until end of February 2022 followed by BA.2. The infection rate for a median of 21 days after PrEP was only 3% in the kidney transplant patients. However, other studies reported the median time to infection from PrEP to be roughly three months, so the shorter observation period in the French study may have biased the results towards ‘over-effectiveness’.

A very recent paper from Normandy, France nicely summarised the current understanding and likely consensus on cilgavimab/tixagevimab PrEP in kidney transplant vaccine non-responders.^19^ The study protocol and time/duration of the study was similar to ours and thus data may be comparable. From January to September 2022 112 kidney graft recipients received PrEP with 2 × 150 mg cilgavimab/tixagevimab and were less likely to develop a COVID-19 infection in the region of Rouen in the Normandy. Based on these data the authors concluded that their PrEP was effective for the prevalent BA.2 and some BA.5 variants. Still, the continued mutation of the spike protein renders cilgavimab/tixagevimab ineffective and reinforces the need for continuous adaptation and generation of new antibodies for SARS-CoV-2.^20^ Therefore, many colleagues stress the importance of performing regular genomic surveillance to identify new resistant SARS-CoV-2 variants as early as they appear and to perform molecular characterization of cilgavimab/tixagevimab and others in live-virus *in vitro* neutralization tests of SARS-CoV-2 variants.^21^

Given the great spectrum of PrEP studies in immunocompromised patients, a meta-analysis would be a good tool to derive an objective and quantitative estimate of the effectiveness of PrEP in this population. However, most of these studies evaluated both therapy and prophylaxis at the same time. We cite here two newer systematic reviews followed by a meta-analysis that reported prophylaxis separately or investigated only prophylaxis in immunocompromised hematology patients.^17^^,^^22^ The drawback of this approach is however the increased heterogeneity resulting in higher variability of the effect estimates. This makes it difficult to estimate the effectiveness of cilgavimab/tixagevimab PrEP in this population. Furthermore, the inclusion of studies from different eras with different omicron variants and cilgavimab/tixagevimab sensitivity limits the comparability to our data.

There are also several treatment studies of cilgavimab/tixagevimab or antiviral drugs in severely sick patients with different comorbidities,.^23^, ^24^, ^25^ Since these studies do not directly apply to the topic of PrEP in immunocompromised transplant patients in the omicron era, we will not go into details about their findings. Our summary of these studies is that cilgavimab/tixagevimab was used off label in severely sick patients as a last resort due to lack of other treatments, but that no clear conclusion can be drawn. It is of note that most of these studies did not report a benefit of an add-on therapy with the antibody combination.

In our study we could show that PrEP with cilgavimab/tixagevimab in kidney transplant recipients led to a lower rate of infections with the omicron subvariant BA.2 but not with BA.5 compared to a matched control group. Our study exhibits some limitations. Patients were not randomised because equipoise, the key prerequisite for a RCT, was not given since *in vitro* neutralization studies showed an effect of the antibody combination against variants at the time of study planning. Given this setting, we cannot formally estimate causal effects, but our study design allows for valid comparisons between the groups through our pairing procedure. The sample size was determined by the available drug supply. Furthermore, given the highly variable incidence rates of infections over time a formal power calculation would have been of low precision. Nevertheless, we were able to match a nearly identical control group in terms of clinical covariables, time of exposure and follow up to our intervention cohort. The dose of 2 × 150 mg was standard at the time of study and a later amendment by Astra Zeneca to use 2 × 300 mg could not be affected, because of the limited drug supply mentioned. However, as mentioned above, most studies in transplant patients did not find good evidence that 2 × 300 mg of cilgavimab/tixagevimab was more effective than the lower dose. We are therefore confident that these potential limitations do not jeopardise the results and conclusions of our study which has several strengths.

In this study, we were able to not only match a very similar control group but also consider the background risk of infection and prevalent SARS-CoV-2 variants over time in the regional population. Since it was mandatory that all regional laboratories report their test results to the central national authority AGES, we could track the infection, the variant and the severity of illness in each case on the background of the general population in Austria. This was a unique chance for our study. The local Department of Virology of the Medical University of Vienna identified the SARS-CoV-2 variant of positive cases and determined the effectiveness threshold for each of the prevalent variants with *in vitro* live-virus neutralization tests. These test results correlated tightly with the clinical courses. Finally, the sequential measurements of the serum concentrations of cilgavimab and tixagevimab allowed for a precise estimate not only of the half-life of these modified antibodies but also of pharmacodynamic efficacy.

Given this unique study setting of almost 400 kidney transplant recipients who were phenotypically characterised in large detail, the centralised national virology test strategy free of charge and the adjacent *in vitro* live-virus neutralization tests we are confident that our results are valid. We conclude that in prevalent kidney transplant recipients who failed immunization after SARS-CoV-2 vaccinations in the months prior to the study and who did not exhibit a prior COVID-19 infection, cilgavimab/tixagevimab PrEP was associated with less omicron BA.2 infections after PrEP administration.

## Contributors

All authors read and approved the final version of the manuscript. Andreas Heinzel, Roman Reindl-Schwaighofer and Rainer Oberbauer accessed and verified the underlying data. Roman Reindl-Schwaighofer: study design and protocol, funding acquisition, study administration, patient specific management, data interpretation, paper writing. Andreas Heinzel: data collection, data curation and statistical analysis, figures and tables. Lukas Raab: data collection and project administration, paper review. Robert Strassl: data collection, data interpretation, paper review. Carsten Herz: patient management, data collection, paper review. Florina Regele: patient management, data collection, paper review. Konstantin Doberer: patient management, data collection, paper review. Oliver Helk: patient management, data collection, paper review. Paul Spechtl: patient management, data collection, paper review. Constantin Aschauer: patient management, data collection, paper review. Karin Hu: data collection, data curation and papa review. Rahel Jagoditsch: patient administration, data collection and curation and paper review. Bianca Reiskopf: patient administration, data collection and curation and paper review. Georg Böhmig: patient management, data collection, paper review. Bernhard Benka: data collection, data interpretation, paper review. Benedikt Mahr: study management, funding acquisition, paper review. Karin Stiasny: data collection, data interpretation, paper review. Lukas Weseslindtner: data collection, data interpretation, paper review. Michael Kammer, data analysis, paper review. Thomas Weckerle: study management, funding acquisition, paper review. Rainer Oberbauer: study design and protocol, obtained funding, paper writing, supervision.

## Data sharing statement

Deidentified data and related documents of this cohort study may be shared upon request with academic research colleagues after the paper has been accepted for publication. Each request will be evaluated and decided by the study board members. Please contact the corresponding author for further information.

## Declaration of interests

“The authors declare no competing interests for this manuscript”
