# Supplementary material for: Cilgavimab and tixagevimab as pre-exposure prophylaxis in vaccine non-responder kidney transplant recipients during a period of prevalent SARS-CoV-2 BA.2 and BA.4/5 variants—a prospective cohort study (RESCUE-TX)
Source: eBioMedicine. 2024 Oct 22;109:105417. doi: 10.1016/j.ebiom.2024.105417 (PMC11539723; doi:10.1016/j.ebiom.2024.105417)
Supplement: Supplementary Figs. S1 and S2 and Tables S1–S3 [file mmc1.docx]

Figure S1

Forest plot showing standardized estimates of the multivariable linear model for peak antibody level at two weeks.


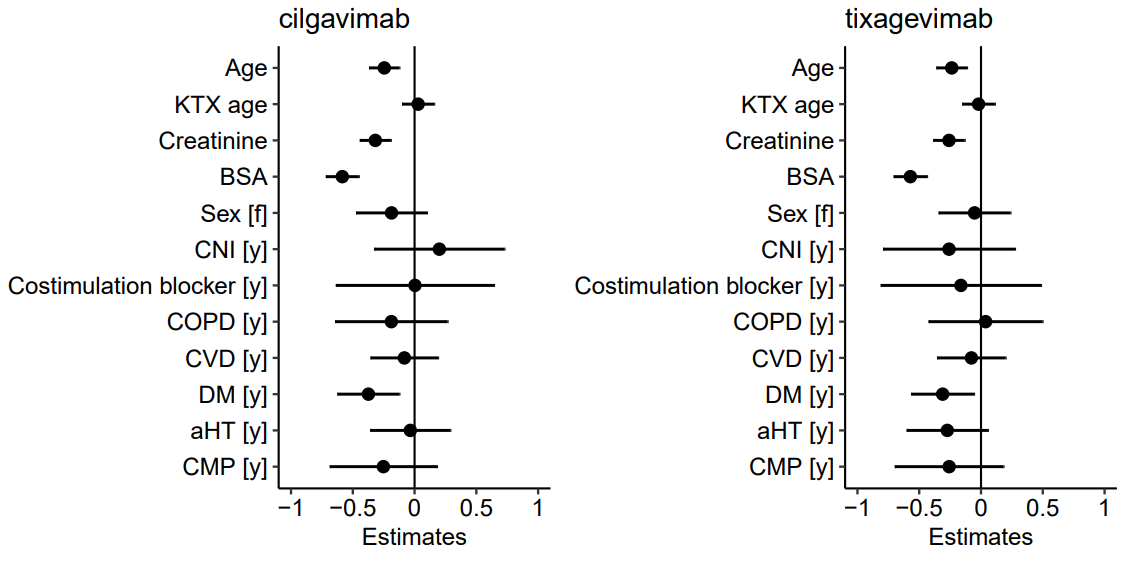


Figure S2

Serum concentration of cilgavimab (left panel) and tixagevimab (right panel) over 48 weeks. The grey line indicates a smoothed estimate for concentration levels from linear mixed models, starting at the peak concentration level two weeks after PrEP. The blue line represents the smoothed estimate for concentration levels of a fully adjusted for clinical covariables linear mixed model. The overlap of both lines indicates no influence of clinical covariables on the antibody decline.


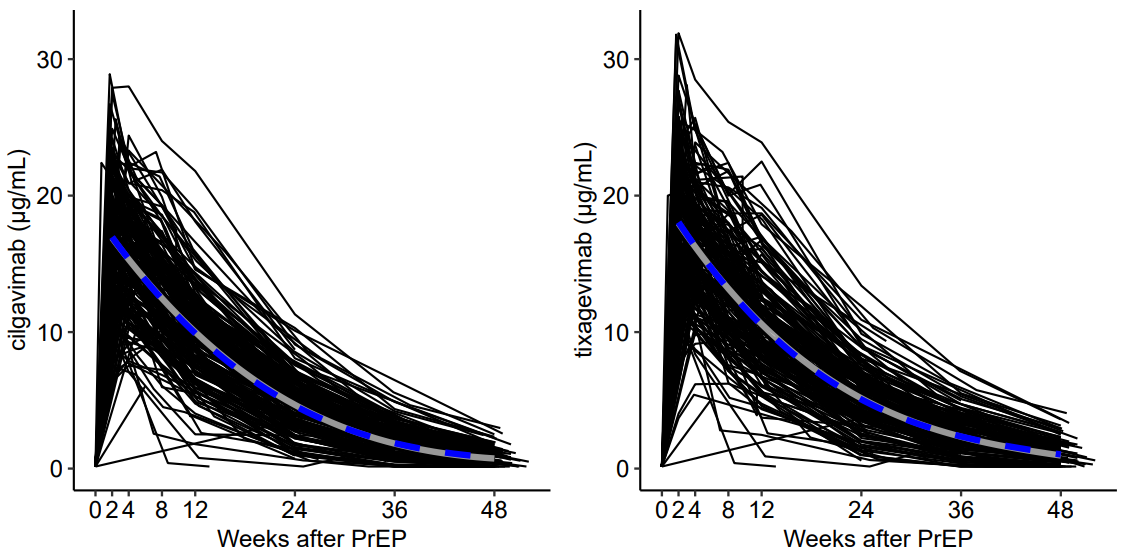


Table S1

Geometric mean and SD of antibody levels at the study visits

| visit | cilgavimab | | tixagevimab | |
| --- | --- | --- | --- | --- |
|  | geometric mean | geometric SD | geometric mean | geometric SD |
| BL | 0.2 | 1.0 | 0.2 | 1.1 |
| W2 | 15.9 | 1.3 | 17.0 | 1.4 |
| W4 | 14.8 | 1.3 | 15.5 | 1.3 |
| W8 | 11.9 | 1.5 | 12.4 | 1.5 |
| W12 | 8.6 | 1.7 | 9.2 | 1.7 |
| W24 | 3.8 | 1.8 | 4.3 | 1.9 |
| W36 | 1.6 | 2.1 | 2.0 | 2.1 |
| W48 | 0.7 | 2.4 | 0.9 | 2.5 |

Table S2

Number of SARS-CoV-2 infections in the PrEP-group per week. For more than half of the SARS-CoV-2 infections the variant was determined by RT-PCR melting-curve, and thus, the number of infections are provided stratified by variant where available. In addition, the percentages of variants detected in the SARS-CoV-2 mutation screening program carried out at the Medical University of Vienna are provided to allow for a better understanding of regional dominant variants throughout the reporting period. After the phase of high SARS-CoV-2 incidence driven by BA.2, the first BA.4/5 variants appeared in early May of 2022 during a phase of overall low SARS-CoV-2 incidence (see figure 4 and figure 5B).

|  | **RESCUE-TX N pos = 35 N typed = 18** | | | | **Medical University of Vienna N pos = 30,092 N typed = 7,328** | | | |
| --- | --- | --- | --- | --- | --- | --- | --- | --- |
| **Week** | **#BA.1** | **#BA.2** | **#BA.4/5** | **#not typed** | **%Delta/Kappa** | **%BA.1** | **%BA.2** | **%BA.4/BA.5** |
| 3/1/2022 |  |  |  |  |  | 30.71 | 69.29 |  |
| 3/7/2022 |  |  |  |  |  | 26.25 | 73.75 |  |
| 3/14/2022 |  | 1 |  |  |  | 15.46 | 84.54 |  |
| 3/21/2022 |  | 1 |  |  |  | 9.20 | 90.80 |  |
| 3/28/2022 | 1 | 1 |  | 1 |  | 4.28 | 95.72 |  |
| 4/4/2022 |  | 3 |  | 1 |  | 2.97 | 97.03 |  |
| 4/11/2022 |  | 1 |  | 3 |  | 2.22 | 97.78 |  |
| 4/18/2022 |  |  |  |  |  | 2.37 | 97.63 |  |
| 4/25/2022 |  | 1 |  |  |  | 2.68 | 97.32 |  |
| 5/2/2022 |  |  |  |  |  | 0.50 | 98.50 | 1.00 |
| 5/9/2022 |  |  |  | 1 |  | 2.91 | 94.17 | 2.91 |
| 5/16/2022 |  | 1 |  | 1 |  |  | 89.29 | 10.71 |
| 5/23/2022 |  | 1 |  |  |  |  | 83.33 | 16.67 |
| 5/30/2022 |  |  |  |  |  |  | 67.69 | 32.31 |
| 6/6/2022 |  |  |  |  |  |  | 43.51 | 56.49 |
| 6/13/2022 |  |  |  |  |  |  | 24.34 | 75.66 |
| 6/20/2022 |  |  | 2 | 2 |  |  | 11.61 | 88.39 |
| 6/27/2022 |  |  |  | 1 | 0.30 |  | 7.23 | 92.47 |
| 7/4/2022 |  |  |  | 1 |  | 1.21 | 5.15 | 93.64 |
| 7/11/2022 |  | 1 |  | 2 |  |  | 1.98 | 98.02 |
| 7/18/2022 |  |  | 1 | 2 |  |  | 1.41 | 98.59 |
| 7/25/2022 |  |  | 1 |  |  |  | 3.57 | 96.43 |
| 8/1/2022 |  |  | 1 |  |  | 1.01 | 2.01 | 96.98 |
| 8/8/2022 |  |  | 1 | 2 |  |  |  | 100.00 |

Table S3

Summary of uOR (unadjusted) and aOR (adjusted) for efficacy outcomes of PrEP

| outcome | type | OR | lower | upper |
| --- | --- | --- | --- | --- |
| infection incidence over 24 weeks follow-up period | uOR | 0.62 | 0.38 | 1.00 |
|  | aOR | 0.66 | 0.38 | 1.12 |
|  |  |  |  |  |
| infection incidence until mid-May | uOR | 0.35 | 0.18 | 0.66 |
|  | aOR | 0.33 | 0.16 | 0.68 |
|  |  |  |  |  |
| infection incidence after mid-May | uOR | 1.31 | 0.63 | 2.72 |
|  | aOR | 1.40 | 0.64 | 3.12 |
|  |  |  |  |  |
| symptomatic infection incidence until mid-May | uOR | 0.37 | 0.17 | 0.79 |
|  | aOR | 0.38 | 0.16 | 0.86 |
|  |  |  |  |  |
| symptomatic infection incidence after mid-May | uOR | 0.96 | 0.43 | 2.15 |
|  | aOR | 1.10 | 0.46 | 2.65 |
